# The Generation of Insects in the Alimentary Canal

**Published:** 1861-04

**Authors:** 


### EDITORIAL AND MISCELLANEOUS.

### The Generation of Insects in the Alimentary Canal.

Our readers will remember the report of a case of epilepsy by Dr. G. W. Powell, of Childensburg, Alabama, a few months since, in which was exhibited the curious phenomenon of insects being discharged from the stomach and bowels. This state of things seems still to exist, as we have received another lot of the two varieties of little animals, insects and worms; both seem to be of the same kinds as sent before, but some of them somewhat larger and opake, and consequently less adapted to microscopical examination. The following extracts from the letter, accompanying the last specimens, will show that they are continually generated in the alimentary canal:

"Inclosed you will find a few more parasitic animals, which I send you for examination. These bugs were passed off by the bowels. I want their microscopical description, if you will be so kind as to send it. I will keep notes of the case, together with your account of their appearance when examined with the microscope, and when sufficiently noted, will write out for publication. I am giving the young man medicine again, and since he commenced its use the parasites are passing by the bowels altogether.

"I need not give you any particulars of the case from which the parasites are obtained at this time, but will do so in future. One of these bugs lived some three or four days after its expulsion from the bowels, and perhaps might have lived as many weeks, had I not confined it in too warm a place. Is it common for any parasitic animal to have antennæ?"

We hope to be able to furnish our readers a full account of this anomalous case; the progress of the case in regard to the convulsions, as well as the generation and discharge of these pecalian insects and worms.

